# Levosimendan in Cardiogenic Shock: A Review of Mechanisms, Clinical Evidence, and Therapeutic Use

**DOI:** 10.31083/RCM47062

**Published:** 2026-05-21

**Authors:** Stefano Salvini, Valeria Trivelloni, Ilenia Monaco, Fatima Samet Bouhaik, Insaf Chouarfia, Iheb Guefrachi, Yassine Bencharef, Mounia Sedrati, Charaf Mouhand, Dario Bottigliero

**Affiliations:** ^1^Department of Cardiology, Centre Hospitalier Général Victor Jousselin de Dreux, 28100 Dreux, France

**Keywords:** levosimendan, cardiogenic shock, heart failure, infusions intravenous, extracorporeal membrane oxygenation, ventricular assist devices, catecholamines, phosphodiesterase inhibitors, troponin C, vasodilation

## Abstract

Levosimendan is a calcium-sensitizing inodilator that has attracted renewed attention for a potential role in the management of cardiogenic shock (CS). The pharmacological profile of levosimendan differs markedly from that of adrenergic inotropes: levosimendan augments contractile force without increasing intracellular calcium or myocardial oxygen demand and, through activation of ATP-sensitive (KATP) potassium channels, produces systemic and coronary vasodilation. Experimental and clinical data also suggest additional protective effects, including modulation of inflammatory pathways, anti-apoptotic activity, and improved mitochondrial function. Although these mechanisms translate into consistent hemodynamic improvement across several studies, large, randomized trials have not demonstrated a consistent survival advantage, likely due to differences in patient selection, treatment timing, and concomitant therapies. Nevertheless, certain clinical groups, such as patients who fail to respond to catecholamines, individuals on chronic β-blockers, and selected perioperative or mechanically supported patients, appear more likely to benefit. Therefore, current guidance favors an individualized rather than universal approach to levosimendan use. Several ongoing investigations, including trials in extracorporeal membrane oxygenation (ECMO)-supported patients and those with septic cardiomyopathy, may help clarify the optimal indications and timing for levosimendan use. This review integrates mechanistic, clinical, and safety data to identify patient profiles most suited to levosimendan therapy and to outline areas where further study is needed.

## 1. Introduction

Cardiogenic shock (CS) represents the final and most severe stage of cardiac pump failure, in which a critical reduction in cardiac output leads to inadequate tissue perfusion and rapid clinical deterioration. Although acute myocardial infarction (AMI) has traditionally been considered the predominant cause, contemporary data from cardiac intensive care units (CICUs) indicate a progressive epidemiological shift. Episodes of CS related to acute-on-chronic heart failure (HF-CS) are now encountered more frequently than AMI-associated cases, reflecting changes in population aging, heart failure prevalence, and patterns of acute cardiac care [1, 2, 3, 4].

Importantly, CS is not a uniform clinical entity. According to contemporary consensus definitions proposed by the Shock Academic Research Consortium, CS can be broadly categorized into four major groups: MI-related cardiogenic shock, acute or decompensated heart failure–related cardiogenic shock, post-cardiotomy cardiogenic shock, and cardiogenic shock due to mechanical complications or less common causes [5]. In parallel, severity-based staging systems, such as the SCAI shock classification, have highlighted the dynamic and heterogeneous nature of CS, with substantial variability in haemodynamic profiles, organ dysfunction, and short-term prognosis across patients [6, 7].

This heterogeneity has important therapeutic implications. Findings derived from one CS phenotype or severity stage cannot be automatically extrapolated to others, and uniform treatment strategies may fail to address the underlying pathophysiology in individual patients. Contemporary registry data and expert consensus statements increasingly emphasize a phenotype-driven approach. Management is guided by physiology, haemodynamics, end-organ function, and responsiveness to pharmacological and mechanical support [8, 9].

Within this complex clinical landscape, the role of inotropic and inodilator therapies remains a subject of ongoing debate. Levosimendan, a calcium sensitizer with additional vasodilatory and cytoprotective properties, has been extensively investigated across different settings of acute and advanced heart failure and, to a lesser extent, in CS. However, its clinical effects appear to vary according to shock etiology, disease severity, and treatment context. A nuanced appraisal of the available evidence is therefore required to define the appropriate role of levosimendan within contemporary, individualized CS management.

## 2. Methods

**Fig. 1.**
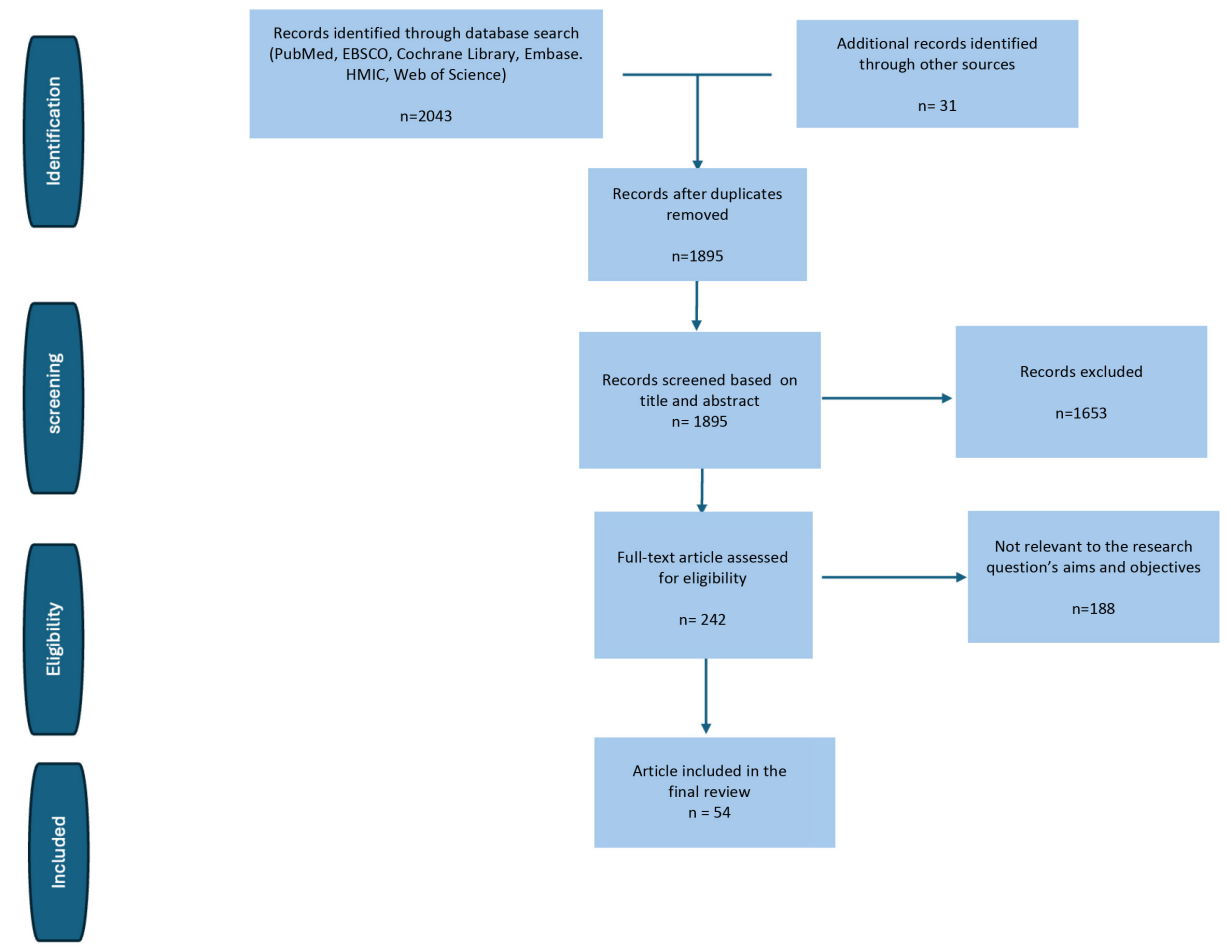
**Simplified PRISMA-like flow diagram illustrating the study identification and selection process used in this narrative review**.

This review was developed as a narrative synthesis of available evidence. To ensure a comprehensive overview, we carried out structured searches in PubMed, ClinicalTrials.gov, and the European Society of Cardiology document repository, without applying date restrictions. Eligible studies included randomized trials, observational cohorts, meta-analyses, expert statements, and perioperative or extracorporeal membrane oxygenation (ECMO)-related reports, provided they were written in English and involved adult patients. We excluded case reports, non–peer-reviewed material, and publications not directly related to heart failure or cardiogenic shock. Titles and abstracts were screened first, followed by full-text review of potentially relevant articles. As this is not a systematic review, no formal risk-of-bias tools were applied, and the PRISMA methodology was not used. For clarity, a simplified flow diagram summarizing the identification and selection process is presented in Fig. 1.

## 3. Results

### 3.1 Mechanism of Action

**Fig. 2.**
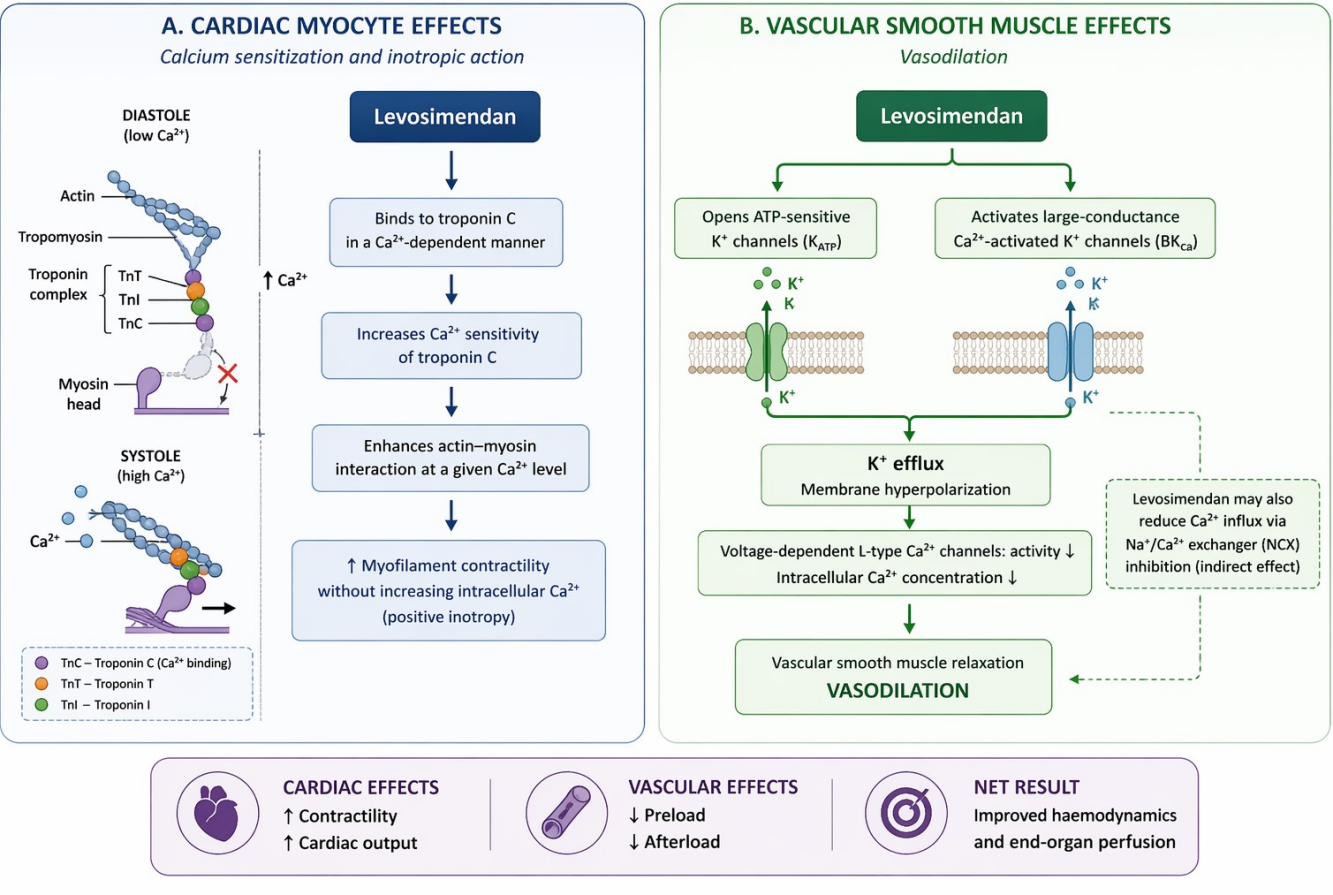
**Mechanistic actions of levosimendan on myocardial contractility and vascular tone**. (A) Levosimendan enhances myocardial contractility by binding to the Ca^2+^-saturated form of cardiac troponin C during systole, thereby stabilizing the contractile apparatus and promoting actin–-myosin interaction without increasing intracellular Ca^2+^ concentration. Diastolic relaxation is preserved. (B) In vascular smooth muscle, levosimendan induces vasodilation primarily through activation of ATP-sensitive (KATP) and other potassium channels, leading to membrane hyperpolarization, reduced Ca^2+^ influx, and subsequent vascular relaxation. Additional modulation of L-type Ca^2+^ channels and Na^+^/Ca^2+^ exchange may further contribute to decreased intracellular Ca^2+^ availability.

Levosimendan (Simdax®, Orion Pharma, Espoo, Finland) acts through a combined mechanism of calcium sensitization and vasodilation, clearly distinguishing it from conventional adrenergic inotropes. By stabilizing the calcium–troponin C interaction, it enhances systolic performance without increasing intracellular calcium concentrations or impairing diastolic relaxation, thereby avoiding the rise in myocardial oxygen consumption typically associated with catecholamines [10, 11]. In parallel, levosimendan opens ATP-sensitive (KATP) potassium channels on both sarcolemmal and mitochondrial membranes, promoting systemic and coronary vasodilation and reducing cardiac preload and afterload [11, 12]. A schematic representation of these mechanisms is shown in Fig. 2 (Ref. [13]).

These properties help explain why levosimendan is associated with a lower incidence of arrhythmias and does not increase myocardial oxygen demand, making it particularly attractive in patients receiving chronic β-blocker therapy or with reduced responsiveness to catecholamines [14, 15]. Experimental data further support mitochondrial stabilization and modulation of inflammatory and apoptotic pathways, suggesting a broader cytoprotective profile beyond pure inotropic support [11, 15].

Clinical findings are consistent with this physiological framework. A meta-analysis of randomized controlled trials demonstrated improvements in cardiac output, pulmonary capillary wedge pressure, and natriuretic peptide levels without a parallel increase in adverse events [16]. Despite its short plasma half-life, the active metabolite OR-1896 confers a prolonged haemodynamic effect lasting several days after infusion, which may contribute to sustained clinical benefit [17].

Overall, these haemodynamic and pleiotropic effects have been comprehensively summarized in mechanistic studies, clinical trials, expert reviews, and consensus-oriented updates [18, 19, 20, 21, 22].

### 3.2 Safety and Dosing Considerations

Safety considerations differ across CS phenotypes, and findings from HF-CS or post-cardiotomy settings cannot be uncritically applied to acute myocardial infarction–related cardiogenic shock (AMI-CS). Contemporary registry data further highlight substantial clinical and haemodynamic heterogeneity between de novo and acute-on-chronic presentations of HF-related CS, with important implications for inotrope selection and tolerability [23]. Over more than two decades of clinical use, levosimendan has been generally well tolerated. Hypotension and tachycardia remain the most frequent adverse events, especially when a loading dose is administered. In hypotensive patients, avoiding the bolus and initiating a low-dose continuous infusion (e.g., 0.05 µg/kg/min) is typically preferred [12]. Notably, the drug has not been associated with excess arrhythmias or renal deterioration, even when used with vasopressors [11]. Its non-adrenergic mechanism also supports use in haemodynamically fragile patients and those receiving β-blockers.

#### 3.2.1 Comparisons With Catecholamines

The consensus statement by Farmakis *et al*. [24] endorses a pragmatic, phenotype-driven selection of inotropes. Within this framework, levosimendan is favored in patients on chronic β-blockers, in those prone to arrhythmias, and in individuals with catecholamine-refractory low output. Unlike traditional adrenergic agents, levosimendan does not induce tachyphylaxis and does not increase myocardial oxygen demand, as its action does not rely on β-receptor stimulation [11, 14, 25].

#### 3.2.2 Comparison With PDE-3 Inhibitors

Levosimendan has also been compared with phosphodiesterase-3 inhibitors such as milrinone. Preclinical studies suggest that levosimendan enhances myocardial contractility with a relatively smaller increase in oxygen consumption, indicating a more favorable energetic profile [26]. Clinical evidence is less uniform. Perioperative studies show that both agents improve haemodynamics, without a consistent advantage of one over the other [27]. In acute heart failure patients with renal impairment, observational data indicate comparable overall efficacy, although hypotension and arrhythmias appear more frequent with milrinone, in line with its cAMP-mediated mechanism [28]. Recent narrative reviews conclude that levosimendan may provide greater haemodynamic stability in hypotensive or adrenergic-intolerant patients, whereas milrinone remains useful when rapid titration is required [29]. Overall, these findings support an individualized approach, guided by haemodynamic profile, arrhythmic risk, and renal function.

#### 3.2.3 Additional Comparative Evidence

Post-hoc analyses from SURVIVE showed significantly lower early mortality in β-blocked patients treated with levosimendan compared with dobutamine (1.5% vs. 5.1%) [30]. In patients with acute decompensated heart failure and renal dysfunction, levosimendan improved measured glomerular filtration rate (GFR) and cardiac output more effectively than dobutamine [31]; observational studies also found higher ejection fraction, shorter ICU stays, and a trend toward lower mortality [32]. A trial-sequential meta-analysis suggested a possible mortality benefit (odds ratio (OR) 0.45; 95% confidence interval (CI) 0.24–0.84), although the cumulative sample size was insufficient for firm conclusions [33]. In sepsis-related shock, available meta-analyses do not show consistent survival advantages compared with other inotropes [34]. A network meta-analysis in CS ranked levosimendan favorably for safety, although efficacy estimates remained limited by substantial study heterogeneity [35]. A recent European position document recommends levosimendan as a short-term option in acute heart failure unresponsive to standard therapy and advises avoiding bolus dosing in hypotensive patients [36].

### 3.3 Clinical Evidence of Levosimendan Across CS Phenotypes

#### 3.3.1 Heart Failure–Related Cardiogenic Shock (HF-CS)

Among the different cardiogenic shock phenotypes, HF-CS is the one in which levosimendan has been examined most thoroughly. In patients with acute or advanced heart failure and low-output states, large, randomized trials such as REVIVE I–II and SURVIVE consistently documented early haemodynamic and symptomatic improvement—lower B-type natriuretic peptide (BNP) concentrations, relief of dyspnea, and better short-term clinical status. None of these benefits, however, translated into a reproducible mortality reduction [30, 37].

Some of the heterogeneity in outcomes likely reflects differences in baseline physiology and treatment context. Notably, in the SURVIVE trial, patients receiving chronic β-blockers appeared to derive an early survival advantage with levosimendan over dobutamine, supporting a phenotype-specific effect. Additional randomized and observational studies have described improved renal indices, higher measured GFR, and shorter ICU stays in patients with concomitant renal impairment—another subgroup that may benefit from the drug’s non-adrenergic profile [31]. Meta-analyses broadly confirm stronger haemodynamic effects compared with adrenergic inotropes and suggest a possible survival signal in selected HF-CS populations, although limitations in sample size and study heterogeneity preclude firm conclusions [33, 38].

#### 3.3.2 Post-Cardiotomy Cardiogenic Shock (PCCS)

The perioperative setting represents a major context in which levosimendan has been extensively investigated. Randomized trials assessing its preventive or therapeutic use in patients undergoing cardiac surgery with impaired ventricular function have yielded mixed results. The levosimendan in patients with left ventricular dysfunction undergoing cardiac surgery (LEVO-CTS) trial, which enrolled patients with a left ventricular ejection fraction ≤35%, demonstrated modest improvements in haemodynamic parameters but failed to show a significant reduction in the composite endpoint of mortality, myocardial infarction, or need for mechanical circulatory support [39].

Consistent findings were reported in the large multicentre trial by Landoni *et al*. [40], in which levosimendan improved selected physiological variables without conferring a survival benefit or reducing major postoperative complications.

Beyond haemodynamic outcomes, a meta-analysis of randomized controlled trials focusing on cardiac surgery populations showed a lower incidence of postoperative acute kidney injury with levosimendan, particularly among high-risk patients, although no consistent effect on mortality was observed [41]. These data suggest a potential organ-protective effect in the perioperative setting that does not translate into a clear survival advantage.

Taken together, current evidence does not support the routine use of levosimendan in unselected cardiac surgical patients. However, it does not exclude a role in carefully selected high-risk PCCS profiles. Patients with postoperative right-ventricular dysfunction, pulmonary hypertension, or inadequate response to catecholamines may derive greater benefit, as the drug’s inodilatory and non-adrenergic properties directly address haemodynamic patterns commonly encountered in these conditions.

Post-cardiotomy evidence has been further contextualized by post-trial clinical updates and perioperative meta-analyses, which emphasize individualized use rather than systematic administration [42, 43, 44, 45].

#### 3.3.3 Acute Myocardial Infarction–Related Cardiogenic Shock (AMI-CS)

Evidence supporting the use of levosimendan in AMI-related CS remains limited. Most large randomized trials conducted in acute decompensated heart failure or perioperative settings explicitly excluded patients with AMI-CS. Consequently, evidence derived from HF-CS or post-cardiotomy populations cannot be directly extrapolated to the acute ischemic setting [30, 37, 39, 40].

AMI-CS is characterized by rapid clinical deterioration driven by ongoing myocardial ischemia. Its haemodynamic profile differs substantially from that of chronic or postoperative cardiac dysfunction. These pathophysiological differences limit the applicability of non-ischemic shock data.

Current expert consensus statements do not recommend routine levosimendan use in AMI-CS [2, 8]. Its administration may be considered only in selected situations, such as persistent low-output states after successful revascularization, intolerance or poor response to catecholamines, or concomitant right ventricular failure, where a non-adrenergic mechanism may offer physiological advantages [11, 36].

These cautious recommendations are consistent with network meta-analyses showing a favourable safety profile for levosimendan but uncertain efficacy in heterogeneous CS populations [35].

#### 3.3.4 Mixed or Undifferentiated CS Populations

Several network meta-analyses have included mixed CS cohorts in which the underlying etiology was not clearly defined. In these analyses, levosimendan often ranked favourably in terms of safety. However, efficacy signals were modest and highly sensitive to between-study heterogeneity [35]. Such data are useful for pharmacological comparisons but should be interpreted cautiously when applied to specific shock phenotypes.

#### 3.3.5 Levosimendan in the Context of ECMO Weaning

Weaning from veno-arterial extracorporeal membrane oxygenation (VA-ECMO) represents a particularly vulnerable phase. It is frequently marked by transient ventricular dysfunction, increased afterload, and ongoing catecholamine dependence.

In this setting, levosimendan has been investigated as an adjunctive therapy to support myocardial performance during the transition from mechanical to native cardiac support. Its non-adrenergic inotropic action, vasodilatory effects, and prolonged haemodynamic activity mediated by its active metabolite provide a clear physiological rationale. This concept was formalized in the design of the LEVOECMO trial [46].

The randomized LEVOECMO trial, recently published in JAMA, did not demonstrate a significant increase in successful VA-ECMO weaning compared with standard care, despite early administration and full-dose levosimendan. These findings indicate that levosimendan does not exert a passive or automatic effect on ECMO weaning. Nevertheless, the trial confirmed an overall acceptable safety profile and provided important haemodynamic information in a highly selected CS population [47].

In parallel, observational data from a propensity score–matched analysis suggested an association between levosimendan use and higher ECMO weaning success rates, although no clear survival benefit was observed [48].

Overall, current evidence supports a cautious and individualized use of levosimendan during VA-ECMO weaning rather than routine administration. This approach is consistent with broader consensus documents on short-term mechanical circulatory support and with observational reports on levosimendan use during extracorporeal support [49, 50, 51].

#### 3.3.6 Summary of Evidence Across Phenotypes

**Fig. 3.**
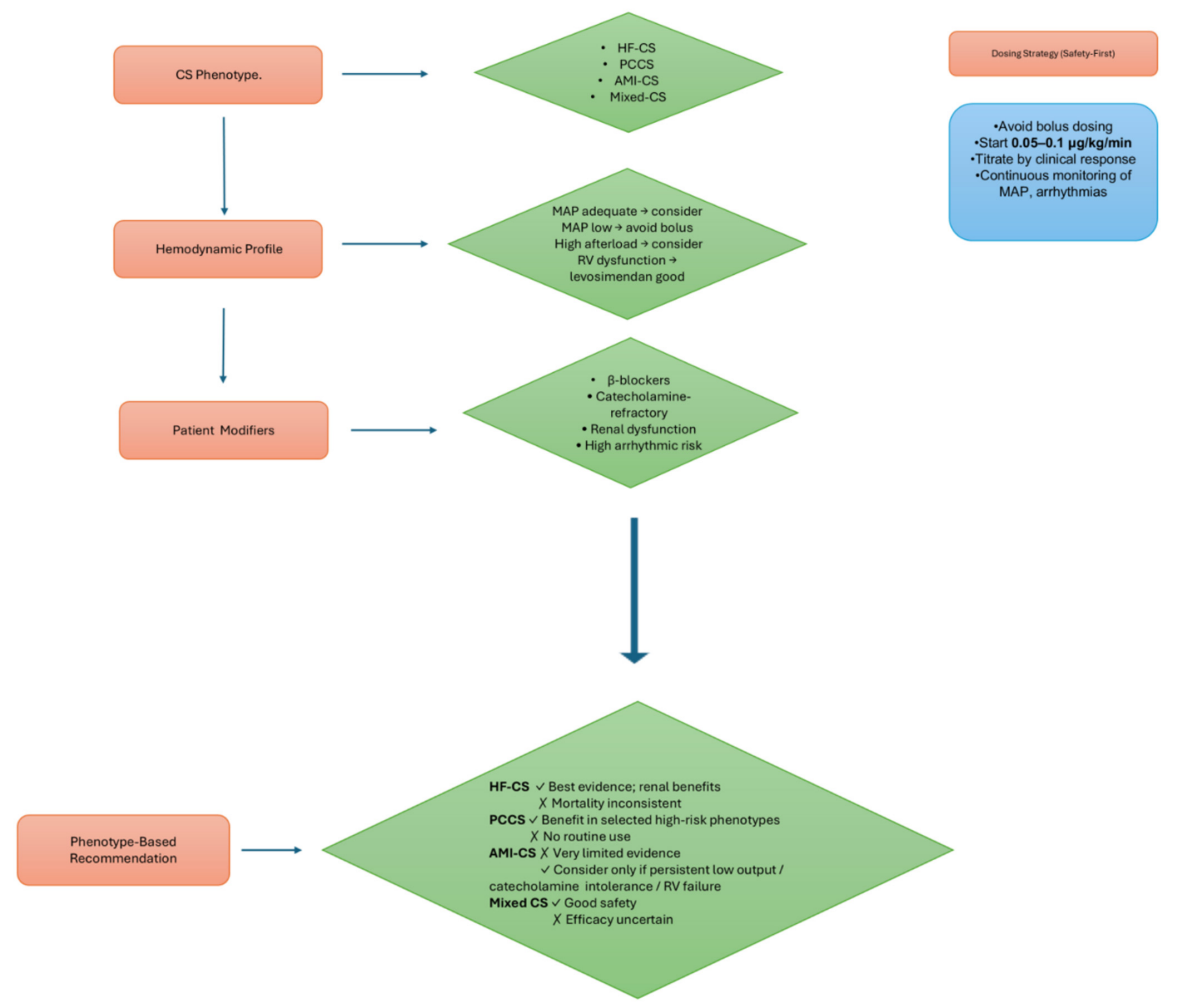
**Phenotype-based guidance for levosimendan use in CS**. Flowchart summarizing phenotype assessment, haemodynamic evaluation, key patient modifiers, and a safety-first dosing strategy to guide individualized levosimendan therapy.

To provide a clearer perspective on the literature, Table 1 (Ref. [30, 31, 34, 35, 37, 38, 39, 40, 52]) compiles the principal clinical studies evaluating levosimendan in acute heart failure and CS. The included trials and cohorts span a broad spectrum of haemodynamic profiles—ranging from HF-CS to PCCS and mixed-etiology shock—and use diverse endpoints, from biomarker trends to hard clinical outcomes. To facilitate interpretation, Table 1 includes a descriptive level of evidence based on study design and sample size. A practical overview of phenotype-guided selection and therapeutic pathways for levosimendan is illustrated in Fig. 3.

**Table 1.** **Summary of key randomized and observational studies evaluating levosimendan across different cardiogenic shock phenotypes**.

Study/Year	Population/CS phenotype	Design & Sample	Main endpoints	Main findings	Level of evidence
Packer *et al*., 2005 (REVIVE II) [37]	Acute decompensated HF (HF-CS/low-output)	RCT	Acute decompensated HF (HF-CS/low-output)	Symptoms, BNP, adverse events, and mortality.	High
Mebazaa *et al*., 2007 (SURVIVE) [30]	Acute decompensated HF with reduced EF	RCT, n = 1327	BNP, 31-day, and 180-day mortality	Greater BNP reduction; early mortality benefit in chronic β-blocker subgroup; no overall 180-day mortality reduction.	High
Lannemyr *et al*., 2018 [31]	HF with renal impairment	RCT	Measured GFR, CO	Levosimendan improved GFR and CO vs. dobutamine.	Moderate
Singh *et al*., 2018 (observational study) [52]	ADHF, reduced EF, renal dysfunction	Observational	EF, ICU stay, mortality	Higher EF, shorter ICU stay, numerical trend toward lower mortality with levosimendan.	Low
Delaney *et al*., 2010 (meta-analysis) [38]	Acute severe HF/inotrope use	Meta-analysis (19 RCTs)	Haemodynamics, mortality	Superior haemodynamic effects; possible survival benefit in some subgroups; no consistent overall mortality reduction.	High
Mehta *et al*., 2017 (LEVO-CTS) [39]	LVEF ≤35% undergoing cardiac surgery (PCCS risk)	RCT	Composite of death, MI, or need for MCS	Modest haemodynamic improvement; no significant outcome benefit.	High
Landoni *et al*., 2017 (post-cardiotomy) [40]	Cardiac surgery patients at risk of low output (PCCS)	Large RCT	Mortality	No mortality benefit; supports selective rather than routine use.	High
Liao *et al*., 2020 (Network meta-analysis) [35]	CS	Network MA	Safety, efficacy ranking	Levosimendan ranks favorably for safety; efficacy signals exploratory.	Low-Moderate
Zangrillo *et al*., 2015 (meta-analysis) [34]	Critically ill adults with sepsis/septic shock	Meta-analysis	Mortality	No consistent mortality benefit.	High

ADHF, acute decompensated heart failure; HF-CS, heart-failure–related cardiogenic shock; PCCS, post-cardiotomy cardiogenic shock; MCS, mechanical circulatory support; VA-ECMO, veno-arterial extracorporeal membrane oxygenation; BNP, B-type natriuretic peptide; NT-proBNP, N-terminal pro-B-type natriuretic peptide; GFR, glomerular filtration rate; CO, cardiac output; RCT, randomized controlled trial; MA, meta-analysis; LEVO-CTS, Levosimendan in patients with left ventricular dysfunction undergoing cardiac surgery. Only studies cited in the manuscript are included.

## 4. Discussion

Across clinical studies, levosimendan consistently improves haemodynamic parameters and end-organ perfusion—effects rooted in its dual action as a calcium sensitizer and vasodilator. Signals of organ protection, particularly involving the kidneys and myocardium, have been observed in physiological studies and in selected groups such as catecholamine-refractory patients, individuals on chronic β-blockers, and those with renal impairment [11, 25, 31, 32, 53].

Most of the evidence derives from HF-related CS and post-cardiotomy cohorts, whereas studies focusing specifically on AMI-CS remain limited. This imbalance means that findings from one phenotype cannot simply be applied to another. Consistent with this, large randomized trials have not demonstrated a uniform survival benefit. Contemporary expert statements therefore view levosimendan primarily as an adjunctive or rescue therapy for high-risk profiles rather than a universal first-line inotrope [8, 9].

In heart failure–related CS, levosimendan repeatedly showed favourable haemodynamic effects, with increases in cardiac output, reductions in pulmonary capillary wedge pressure, and consistent decreases in natriuretic peptides. The REVIVE and SURVIVE trials reported early symptomatic improvement and marked BNP reductions, although these changes did not translate into a clear survival advantage [30, 37]. A post-hoc analysis of SURVIVE indicated a possible early mortality benefit among patients receiving chronic β-blockers [30]. In individuals with concomitant renal dysfunction, levosimendan improved measured GFR and cardiac output more effectively than dobutamine [31], and observational work described higher ejection fractions, shorter ICU stays, and a trend toward lower mortality [52]. Meta-analyses generally confirmed stronger haemodynamic responses than those seen with adrenergic inotropes, while also underscoring the limitations imposed by study heterogeneity and insufficient power to assess mortality [33, 38]. In perioperative settings, including patients at risk for PCCS, the LEVO-CTS trial and other randomized studies documented modest improvements in haemodynamics without reductions in major adverse postoperative outcomes [38, 39]. However, observational cohorts suggest that selected high-risk subgroups, especially those with right-ventricular dysfunction or low-output states refractory to catecholamines, may derive more meaningful benefit.

Evidence in AMI-related CS remains sparse because most pivotal trials excluded this population. Thiele *et al*. [2] emphasize the primacy of rapid revascularization, careful volume management, and individualized consideration of mechanical circulatory support. Current data do not support routine administration in AMI-CS, although patients with persistent low output despite revascularization or those unable to tolerate adrenergic agents may occasionally benefit.

In mixed-etiology cohorts, levosimendan has generally shown a favourable safety profile, while efficacy varied considerably between studies [34]. Experiences reported during COVID-19, associated CS have also shown that levosimendan may contribute to multimodal bailout strategies in highly refractory cases [52].

In patients supported with VA-ECMO, levosimendan has been investigated as an active adjunctive therapy rather than as passive haemodynamic support. However, the randomized LEVOECMO trial, recently published in JAMA, did not demonstrate a significant reduction in time to successful VA-ECMO weaning compared with placebo, despite early administration and full-dose infusion. These findings indicate that levosimendan does not automatically facilitate ECMO weaning. Nevertheless, potential haemodynamic or organ-supportive effects may still be relevant in selected clinical scenarios. Therefore, the use of levosimendan during VA-ECMO weaning should be individualized and not routinely recommended, pending further phenotype-specific analyses and real-world data [46, 47, 48].

The variability in study designs and patient populations underscores a central theme: levosimendan’s clinical impact is strongly phenotype-dependent [23], and improvements in haemodynamics do not automatically translate into uniform survival benefits across all shock categories

### 4.1 Limitations

Interpretation of the available evidence is limited by substantial methodological variability across studies. Differences in patient selection, timing of treatment initiation, dosing strategies, and outcome definitions restrict comparability and reduce the strength of pooled analyses. Many randomized trials lacked adequate power or did not stratify patients according to CS phenotype, making it difficult to determine which groups benefit most. Some clinically important subpopulations—such as patients with STEMI-related shock—remain inadequately represented in the literature. Furthermore, the definitions of clinical response, haemodynamic improvement, and composite endpoints vary widely, complicating the translation of these findings into practice [1, 2].

### 4.2 Future Perspectives

Although levosimendan was first introduced for acute decompensated heart failure, its potential applications have widened over time. It is now being explored in settings characterized by reduced contractile reserve or intolerance to adrenergic stimulation, such as septic cardiomyopathy, isolated right-ventricular failure, or during weaning from temporary mechanical circulatory support, including ECMO [11, 12, 15]. Its pharmacologic profile—calcium sensitization combined with non-adrenergic vasodilation—makes it particularly attractive when β-blockade, renal dysfunction, or arrhythmic risk limit the use of conventional inotropes. In this context, levosimendan is increasingly considered a second-line or adjunctive agent for patients with complex haemodynamic profiles.

A further area of growing interest is perioperative care. Early reports suggest that administration before or around LVAD implantation may improve postoperative stability and reduce weaning failure, supporting broader integration into advanced heart-failure programs [54].

## 5. Conclusions

Levosimendan appears to offer therapeutic value for selected patients with CS, particularly those who respond poorly to catecholamines, are treated chronically with β-blockers, or require support during mechanical circulatory assistance. Its non-adrenergic profile, prolonged pharmacodynamic activity, and favourable safety characteristics make it a suitable option in complex haemodynamic situations.

Despite consistent haemodynamic and organ-protective effects, large randomized trials have not demonstrated a clear survival benefit [30, 37, 39, 40]. More rigorously designed, phenotype-stratified studies are required to define its optimal role. Recent investigations, including the LEVOECMO trial, are expected to clarify patient selection, timing of administration, and overall impact on outcomes [46, 47], paving the way for more individualized and evidence-based use.
